# The Property of a Key Amino Acid Determines the Function of Farnesyl Pyrophosphate Synthase in *Sporobolomyces pararoseus* NGR

**DOI:** 10.3390/cimb46040195

**Published:** 2024-04-03

**Authors:** Yunjiao Wang, Ning Zhang, Jianyu Yan, Chunwang Li, Nan Zeng, Dandan Wang, Zijing Li, Bingxue Li, Yingfeng An

**Affiliations:** 1 College of Bioscience and Biotechnology, Shenyang Agricultural University, Shenyang 110866, China; wyj19970320@163.com (Y.W.); zhangning@syau.edu.cn (N.Z.); yanjianyu718@163.com (J.Y.); lee491867@163.com (C.L.); 2021220160@stu.syau.edu.cn (D.W.); 2 College of Land and Environment, Shenyang Agricultural University, Shenyang 110866, China; zengnan1015@163.com; 3 Food Science College, Shenyang Agricultural University, Shenyang 110866, China; 15002492493@163.com

**Keywords:** farnesyl pyrophosphate synthase, isoprenoids, *Sporobolomyces pararoseus*, carotenoids, site-directed mutation

## Abstract

Farnesyl pyrophosphate synthase (FPPS) catalyzes the synthesis of C15 farnesyl diphosphate (FPP) from C5 dimethylallyl diphosphate (DMAPP) and two or three C5 isopentenyl diphosphates (IPPs). FPP is an important precursor for the synthesis of isoprenoids and is involved in multiple metabolic pathways. Here, farnesyl pyrophosphate synthase from *Sporobolomyces pararoseus* NGR (SpFPPS) was isolated and expressed by the prokaryotic expression system. The *SpFPPS* full-length genomic DNA and cDNA are 1566 bp and 1053 bp, respectively. This gene encodes a 350-amino acid protein with a predicted molecular mass of 40.33 kDa and a molecular weight of 58.03 kDa (40.33 kDa + 17.7 kDa), as detected by SDS-PAGE. The function of SpFPPS was identified by induction, purification, protein concentration and in vitro enzymatic activity experiments. Structural analysis showed that Y90 was essential for chain termination and changing the substrate scope. Site-directed mutation of Y90 to the smaller side-chain amino acids alanine (A) and lysine (K) showed in vitro that wt-SpFPPS catalyzed the condensation of the substrate DMAPP or geranyl diphosphate (GPP) with IPP at apparent saturation to synthesize FPP as the sole product and that the mutant protein SpFPPS-Y90A synthesized FPP and C20 geranylgeranyl diphosphate (GGPP), while SpFPPS-Y90K hydrolyzed the substrate GGPP. Our results showed that *FPPS* in *S. pararoseus* encodes the SpFPPS protein and that the amino acid substitution at Y90 changed the distribution of SpFPPS-catalyzed products. This provides a baseline for potentially regulating SpFPPS downstream products and improving the carotenoid biosynthesis pathway.

## 1. Introduction

Isoprenoids, a family of natural molecules with high chemical diversity, with over 80,000 known isoprenoids [1], are involved in the synthesis of secondary metabolites, such as carotenoids and sterols, in microorganisms, demonstrating promising commercial prospects for industrial production [2,3]. All isoprenoids are derived from a simple C5 structural unit: isoprenyl diphosphate (IPP) and its isomer dimethylallyl diphosphate (DMAPP) [4], which are synthesized from or modified by isoprenyl diphosphate synthases (IPPSs or IDSs), also known as prenyltransferases (PTs) [5]. PTs are involved in the biosynthesis of isoprenoids and regulate the production of secondary metabolites. According to the characteristics of the carbon chain lengths of the biosynthetic products, isoprenyl diphosphate synthases (IPPSs) are classified as follows: short-chain isoprenyl diphosphate synthases (SCIPPSs, C10–C25), medium-chain isoprenyl diphosphate synthases (MCIPPSs, C30–C35) and long-chain isoprenyl diphosphate synthases (LCIPPSs, C40–C50) [6], all of which are classified as trans types, according to the stereochemistry of the double bonds from IPP condensation [7]. By contrast, the product of cis-polypentenyl diphosphate synthase is polyisoprenoid (>C50), which catalyzes the synthesis of a double bond with an all-cis stereoisomeric configuration [8].

Farnesyl pyrophosphate synthase (known variously as FPPS, FPS, FDPS, FDS and EC 2.5.1.10), key enzyme in the metabolic pathway of isoprenoids, belong to the trans-SCIPPS family and catalyze the sequential irreversible 1’-4 condensation of DMAPP with IPPs to synthesize the finial product (2E,6E)-farnesyl diphosphate (FPP, C15). FPPS lead to an electron-deficient substrate carbon atom due to the loss of the diphosphate group and attack of the double-bonded electron-rich carbon atom on the IPP molecule [9]. In 1975, Eberhardt et al. [10] purified a homologous prenyltransferase from *Saccharomyces cerevisiae*, and in 1994 the first X-ray structure of the IPPSs was solved [11]. These results provided a basis to model homologous IPPs found in different species [12].

FPPSs are IPPSs that function as soluble homodimers, with each subunit weighing 32–44 kDa, and exist as single copies in the cytoplasm of microorganisms [13]. The FPPS catalyzing all-trans stereoisomerism consists mainly of α-helices [11], which form a large reaction cavity in the middle of the protein and contain two aspartic acid-rich motifs, designated as the First Asp-Rich Motif (FARM) and the Second Asp-Rich Motif (SARM). These two motifs are typical IPPS active site domains and binding sites for the diphosphate moieties of IPPs and allylic substrates [14]. Previous studies have suggested that the fifth amino acid upstream of FARM is key for FPPS enzymatic activity [15].

*Sporobolomyces pararoseus* NGR is unconventional and can yield high levels of carotenoids [16]. Carotenoids are lipid-soluble isoprenoids with antioxidant activity and are precursors of vitamin A synthesis. Carotenoids are used therapeutically to improve immunity and prevent cancer and many chronic diseases and have great commercial value in cosmetics, as food additives and in the animal feed industries [17,18]. In addition to carotenoids, the functional diversity of terpenoids enables them to play different roles in nature, such as hormones (gibberellins), photosynthetic pigments (phytol and carotenoids) and electronic carriers (ubiquinone and plastid quinone), as well as toxins, insect repellents, etc. [19]. FPPSs are located upstream of the carotenoid biosynthesis pathway. They are responsible for the synthesis of C15 FPP, which is condensed with a molecule of IPP to form C20 GGPP, and via head-to-head condensation of two GGPPs forms the pigment synthesis intermediate C40 phytoene [20]. Changes in FPPS activity affect the flux of FPP to downstream metabolites, which means that the enzyme plays a vital role in regulating downstream metabolites [21,22,23]. Previous studies on FPPSs have been mostly centered on plants and insects, but studies on the encoding of the FPPS gene in pigment-producing fungus have rarely been reported. In this study, the *FPPS* gene from *S. pararoseus* (*SpFPPS*) was successfully isolated and characterized for a better understanding of the role of SpFPPS, the tyrosine 90 (Y90) of FPPS, which plays an important role in affecting the activity of SpFPPS, as is described in this report.

## 2. Materials and Methods

### 2.1. Materials

Unlabeled isopentenyl diphosphate, dimethylallyl diphosphate, geranyl diphosphate, farnesyl diphosphate, geraniol, farnesol and geranylgeraniol were purchased from Sigma-Aldrich (Sigma-Aldrich, St. Louis, MO, USA). All other chemicals were of analytical grade.

### 2.2. Strains and Growth Conditions

*Sporobolomyces pararoseus* NGR was isolated from strawberries [20]. This strain was deposited as CGMCC 2.5280 in the China General Microbiological Culture Collection Center (CGMCC), inoculated in 250 mL Erlenmeyer baffle flasks containing 100 mL of liquid YPD medium (yeast extract 10 g/L, peptone 20 g/L, dextrose 20 g/L, pH 6.0) and cultured in a constant-temperature shaking incubator at 28 °C for 72 h. *Escherichia coli* DH5α and BL21 (DE3) strains (Tiangen, Beijing, China) were cultured in Luria–Bertani (LB) medium (yeast extract 5 g/L, peptone 10 g/L, NaCl 10 g/L, pH 7.0) at 37 °C for 12 h.

### 2.3. Cloning of Genes Encoding the FPPS Enzymes from S. pararoseus NGR

The activated NGR was inoculated in YPD for 72 h and centrifuged at 13,523× *g* for 2 min at 4 °C, and the genomic DNA was extracted using the Rapid Yeast Genomic DNA Isolation Kit (Sangon, Shanghai, China). The total RNA was extracted using the Spin Column Fungal Total RNA Purification Kit (Sangon, Shanghai, China), and cDNA was synthesized using the PrimeScriptTM II 1st Strand cDNA Synthesis Kit (TaKaRa, Dalian, China), according to the manufacturer’s instructions. Genomic DNA and cDNA were stored at −20 °C for subsequent experiments.

We obtained the putative *SpFPPS* (comp9430) sequence from the NGR transcriptome data (register number: SRP131948). Premier 5.0 was used to design specific primers (Table S1), and the restriction enzymes were *Sac*I and *Bam*HI.

The genomic DNA and cDNA of *SpFPPS* in NGR were amplified by 2 × High fidelity PCR mix premix (Sangon, Shanghai, China). The amplification procedure was as follows: 1 cycle of 95 °C for 3 min and 35 cycles of 95 °C for 15 s, 53 °C for 15 s, 72 °C for 1 min and 72 °C for 5 min. The PCR product was purified and ligated into the pMD18-T cloning vector (TaKaRa, Dalian, China) and transformed into *E. coli* DH5α, and the plasmid pMD18-T-*FPPS* was extracted using the Plasmid Extraction Mini Kit (Solarbio, Beijing, China) and sent away for sequencing verification (GeneCreate, Wuhan, China).

### 2.4. Bioinformatic Analysis of Putative FPPS from S. pararoseus

The genomic DNA and cDNA of *SpFPPS* were aligned using DNAMAN to verify exons and introns. ProtParam 3.0 (https://web.expasy.org/protparam/, accessed on 25 February 2024) was used to predict the molecular weight, amino acid composition and theoretical pI of SpFPPS. ProtScale (https://web.expasy.org/protscale/) was used to predict protein hydrophobicity. The transmembrane domain was predicted by the TMHMM Server v.2.0 (https://www.cbs.dtu.dk/services/TMHMM/). The PredictProtein tool (https://www.predictprotein.org/) was used to predict the secondary structure of the SpFPPS protein. SWISS-MODEL (https://www.swissmodel.expasy.org/interactive) was used to construct a 3D model. The three-dimensional structure of the protein was visualized using Pymol (The PyMOL Molecular Graphics System, Version 2.3.0 Schrödinger, LLC, New York, NY, USA).

The FPPS sequences of different species were obtained from the NCBI and compared by the ClustalW algorithm. A phylogenetic tree was constructed by MEGA 11.0 using the neighbor-joining method [24].

### 2.5. Construction of Expression Vectors

pMD18-T-*FPPS* and the expression vector pET-32a (+) were digested with restriction endonuclease *Sac*I and *Bam*HI at 37 °C for 4.5 h. The linearized expression vector pET-32a (+) and the target fragment were recovered and ligated by T4 DNA ligase to construct the recombinant plasmid pET32a-*FPPS*. The plasmids mentioned are shown in Table S2. The ligation product was transformed into *E. coli* DH5α and BL21(DE3), and PCR was used to verify that the plasmid was transformed into *E. coli*. The Plasmid Extraction Mini Kit (Solarbio, Beijing, China) was used to extract plasmids, and the inserted fragments in pET32a-*FPPS* were sequenced to ensure that no mutations were introduced during PCR.

### 2.6. Protein Expression and Purification

*E. coli* BL21(DE3) transformed with pET32a-*FPPS* were inoculated in 50 mL LB medium containing 50 μg/mL Amp at 37 °C for 12 h, and empty pET-32a (+) was used as a control. The cells were inoculated in LB medium at a ratio of 1:25, 1 mM isopropyl β-D-thiogalactoside (IPTG) was added and the cells were cultured at 37 °C for 1–2 h. Protein expression was induced at 20 °C, 23 °C, 26 °C and 28 °C. The cells were cultured for about 4–5 h and centrifuged at 13,523× *g* for 1 min. The cells were washed three times with PBS; then, they were fully suspended with 1.5 mL lysis buffer (50 mM NaH_2_PO_4_, 300 mM NaCl, 10 mM Imidazole, pH 8.0), and 1 mM phenylmethylsulfonyl fluoride (PMSF, Yuanye Bio-Technology, Shanghai, China) was added immediately.

The treated cells were ultrasonically broken for 20 min in 6 s^−1^ pulses with 4 s between pulses on ice (the power was selected at a level slightly below the bubble generation of the solution [40%]). The cell lysate was centrifuged at 14,000× *g* for 30 min at 4 °C, the supernatant was collected in a new tube, the precipitate was fully resuspended with the lysis buffer equal to the supernatant, and the supernatant and the precipitate were both analyzed by sodium dodecyl sulfate polyacrylamide gel electrophoresis (SDS-PAGE, 12% separate gel, 5% concentrated gel) with Coomassie Brilliant Blue staining for their expression. Lightly stained for 1 h with a solution of 25% isopropanol and 10% acetic acid containing 0.1% (*w*/*v*) Coomassie Brilliant Blue R250, they were then decolored with a solution of 10% acetic acid and 5% anhydrous ethanol until the electrophoresis band was clear.

Recombinant protein pET32a-FPPS was purified using a 1 mL HisTrap HP column (Cytiva, Marlborough, MA, USA), and the supernatant obtained was filtered through a 0.22 μm membrane and added to a nickel column to collect the effluent. The wash buffer (50 mM NaH_2_PO_4_, 300 mM NaCl, 20 mM Imidazole, pH 8.0) was filtered through a 0.22 μm filter membrane and added to a nickel column; the enzyme run was performed five times using the Ni column by reloading the effluent, and the effluent was collected separately. The elution buffer (50 mM NaH_2_PO_4_, 300 mM NaCl, 500 mM Imidazole, pH 8.0) was used in the same way as the wash buffer, and the purification of the target protein was analyzed by SDS-PAGE.

### 2.7. Determination of Enzymatic Activity

The target proteins were collected using Amicon Ultra-0.5 Centrifugal Filter (10 kDa) ultrafiltration tubes (Millipore, Billerica, MA, USA), with 100 mM HEPES buffer (contain 5 mM MgCl_2_, pH 7.5) used as the exchange buffer, and the procedure was repeated twice. The collected proteins were concentrated and stored at 4 °C.

In order to verify the reaction product of FPPS, 10 ng FPPS protein, 50 μM IPP and 50 μM substrate (DMAP, GPP, FPP or GGPP) were added to the enzyme activity system, HEPES buffer was added to 200 μL, and the reaction was carried out at 28 °C for 2 h. A quantity of 200 μL 0.2 M Tris-HCl (pH 9.5) buffer containing 2 U Shrimp alkaline phosphatase (SAP, TaKaRa, Dalian, China) and 2 U Thermostable inorganic pyrophosphatase (TIPP, NEB, USA) was added, reacted at 30 °C for 12 h and stopped on ice. This step was performed to dephosphorylate the product of the enzymatic reaction. Then, 400 μL n-hexane was added for extraction, which was performed three times, after which the extract organic fractions were combined and reduced to 100 μL under a stream of nitrogen gas for thin-layer chromatography (TLC) analysis.

All the collected products were analyzed by TLC Silica gel 60 F_254_ (Merck, Darmstadt, Germany). A mixture of geraniol (GOH), farnesol (FOH) and geranylgeraniol (GGOH) was used as the standard, and the mobile phase was toluene/acetonitrile/ethyl acetate/acetic acid (35:5:15:0.15, *v*/*v*/*v*/*v*). The products were visualized using iodine vapor and detected using the Bio-image analyzer KH-3100 (KEZHE, Shanghai, China).

### 2.8. Preparation and Activity Assays of FPPS Mutants

FPPS was mutated by overlap extension PCR. The mutagenic primer sequence is listed in Table S1. Based on the amplification procedure described above, the mutated PCR product was obtained and purified.

The plasmids pMD18-T-*SpFPPS-Y90A* and pMD18-T18-*SpFPPS-Y90K* were constructed and transformed into DH5α. The plasmids pET32a-*SpFPPS-Y90A* and pET32a-*SpFPPS-Y90K* were constructed and transformed into the expression strain BL21(DE3). All mutations were confirmed by DNA sequencing.

The recombinant proteins pET32a-SpFPPS-Y90A and pET32a-SpFPPS-Y90K were induced to express in strain BL21(DE3) at 28 °C and purified by the nickel column. The expression and purification of the mutant recombinant proteins were analyzed by SDS-PAGE.

After the concentration and collection of the mutant recombinant proteins, the reaction products were verified. The enzyme assays contained 10 ng of protein, 50 μM substrate (DMAPP, GPP, FPP or GGPP) and 50 μM IPP as the common substrate at apparent saturation [24], made up to 200 μL by adding HEPES buffer, and the reaction was carried out at 28 °C for 2 h. The IPP product obtained was depyrophosphorylated, extracted by n-hexane, concentrated under nitrogen and stored at −80 °C, as described earlier.

GOH, FOH and GGOH were mixed as standards. All collected products were analyzed by TLC and visualized by iodine vapor. Their distribution was detected by the Bio-image analyzer KH-3100 (KEZHE, Shanghai, China).

## 3. Results

### 3.1. Putative FPPS Gene Cloning and Sequence Analysis

Based on the transcriptome data, specific primers were designed to amplify the *FPPS* gene in NGR, and 1566 bp genomic DNA and 1053 bp cDNA (Figure S1) were obtained and named as SpFPPS.

Genomic DNA and cDNA alignment revealed that the gene contains eight exons and seven introns, with all splicing sites following the GT-AG rule (Figure S2). The nucleic acid sequence and the amino acid sequence of SpFPPS determined in this experiment are available in the GenBank databases under the accession number ON220551.1.

### 3.2. Sequence Analysis of SpFPPS Protein

The molecular weight of SpFPPS was calculated to be 40.33 kDa using the ProtParam 3.0. The theoretical pI value is 5.11, encoding a protein of 350 amino acids. The sequence includes 55 negatively and 42 positively charged amino acids. The protein instability index is 43.55, suggesting that SpFPPS is an unstable protein. ProtScale indicated that the protein is a hydrophilic protein (Figure S3A). The TMHMM Server (v.2.0) predicted that the protein has no transmembrane structure.

The secondary structure of SpFPPS predicted by PredictProtein included α-helixes (63.43%), β-sheets (2.86%), extended strands (7.43%) and random coils (26.29%) (Figure S3B). The main structure is an α-helix, which forms a cavity, and FARM and SARM are located on the two α-helixes opposite. SWISS-MODEL was used to construct a 3D model [25,26]. Based on the prediction of the X-ray and homologous proteins, the SpFPPS model has a sequence similarity of 51.17% with the template protein (PDB:1FPS1); it was visualized using Pymol (Figure S4).

**Figure 1:**
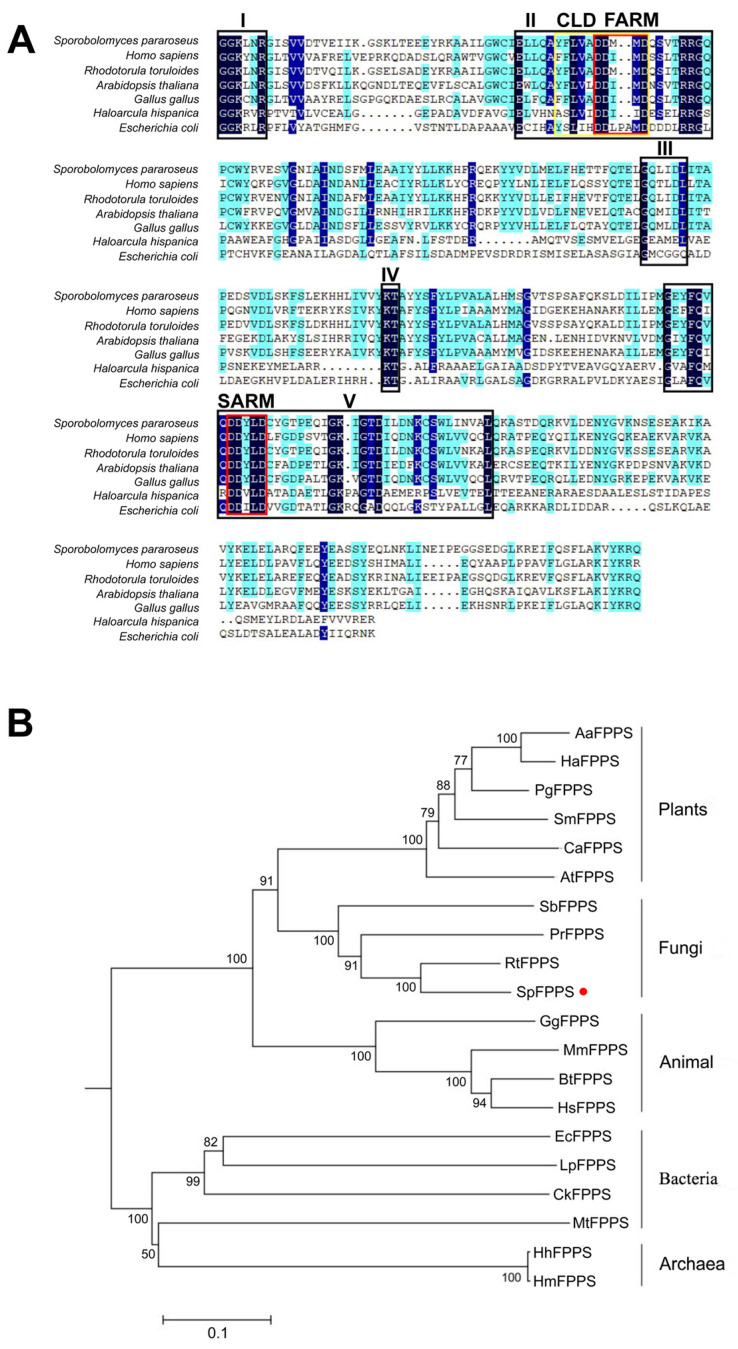
Sequence analysis of FPPS proteins. (**A**) Sequence comparison of FPPSs from seven species. The red box represents FARM and SARM, and the yellow box represents CLD; (**B**) Construction of a phylogenetic tree based on FPPS amino acid sequences of different species using the neighbor-joining method. Red dots marked the SpFPPS in this study.

To further explore the molecular characteristics of the expressed *SpFPPS* gene expression products, we used DNAMAN to compare SpFPPS with FPPSs obtained from different species in the NCBI databank (Figure 1A). The results indicated that the structure contains five conserved regions; regions II and VI are aspartate acid-rich motifs designated FARM and SARM, respectively. The two conserved motifs are located on opposite walls of the central cavity facing each other (Figure S4). The FARM sequence is DDX (2–4) D (where X is any amino acid), which is defined further by upstream residues, while the SARM (DDXXD) is not. The FARM and the upstream five amino acids form a chain-length determination domain (CLD) [27]. According to the CLD sequence, FPPS is divided into three types. Type I FPPS, derived from eukaryote FPPS, has a FARM motif (DDXXD) and two aromatic amino acid residues at the fourth and fifth positions upstream of FARM. Type II FPPS (eubacterial) has a four-amino acid insertion in FARM (DDXXXXD) and a single aromatic amino acid at the fifth site upstream of FARM. Type III FPPS, the FARM motif, has a DDXXD with only one aromatic amino acid that is located at the fifth position [27]. In SpFPPS, a type I FPPS, phenylalanine (F) and Y residues are located at the fourth and fifth positions upstream of FARM, respectively; the X amino acids are M (Figure 1A).

The SpFPPS protein sequence was compared with the FPPS protein sequences of 19 different species identified in the NCBI database to construct a phylogenetic tree. The results showed that the FPPS of *S. pararoseus* had the highest sequence similarity to that of *Rhodotorula toruloides* (79.02%) and belonged to the same group as the FPPSs of fungi (Figure 1B).

### 3.3. Expression and Purification of Recombinant Protein pET32a-SpFPPS

**Figure 2:**
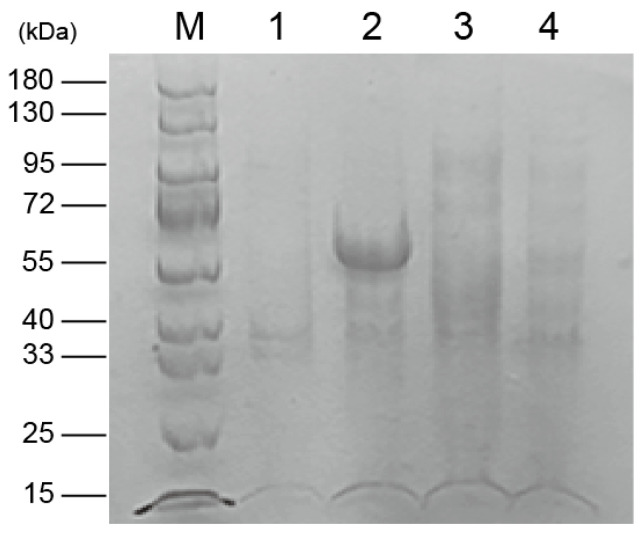
Expression of pET32a-SpFPPS. (M: marker; 1 and 2: supernatants of BL21 (DE3) strains carrying plasmids pET32a and pET32a-SpFPPS after fragmentation; 3 and 4: precipitation of BL21(DE3) strain carrying plasmids pET32a and pET32a-SpFPPS after fragmentation).

**Figure 3:**
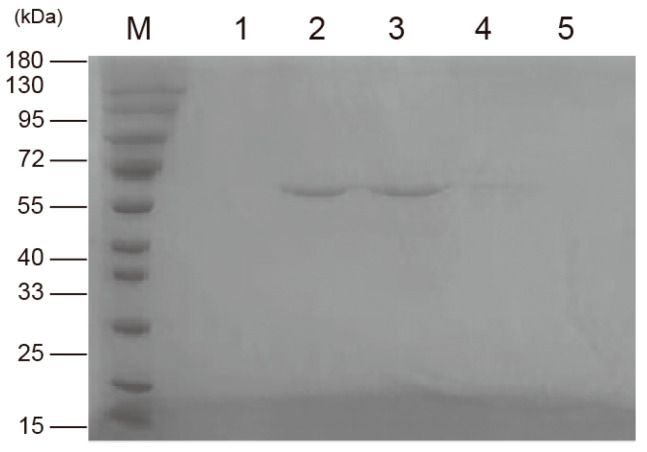
Purification of pET32a-SpFPPS. (M: marker; 1–5: sequential elution with elution buffer).

To characterize SpFPPS, the recombinant plasmid pET32a-SpFPPS was constructed, and the *SpFPPS* gene in *E. coli* was verified by agarose gel electrophoresis. The recombinant protein pET32a-SpFPPS was induced to express at different temperatures. SDS-PAGE analysis showed that the proteins induced at 28 °C were expressed in the supernatant, and the expression level in the precipitate was the lowest and the band was the faintest (Figure S5). pET32a-SpFPPS was induced to express at 28 °C, and the recombinant protein with three tags (one Trx·tag, one His·tag and one S·tag) was obtained. The molecular weight of the recombinant protein was 58.03 kDa (40.33 kDa + 17.7 kDa) (Figure 2). A band of about 60 kDa was eluted from the nickel column purification (Figure 3), indicating that the recombinant protein of the correct size was expressed.

### 3.4. Analysis of In Vitro Activity of SpFPPS

**Figure 4:**
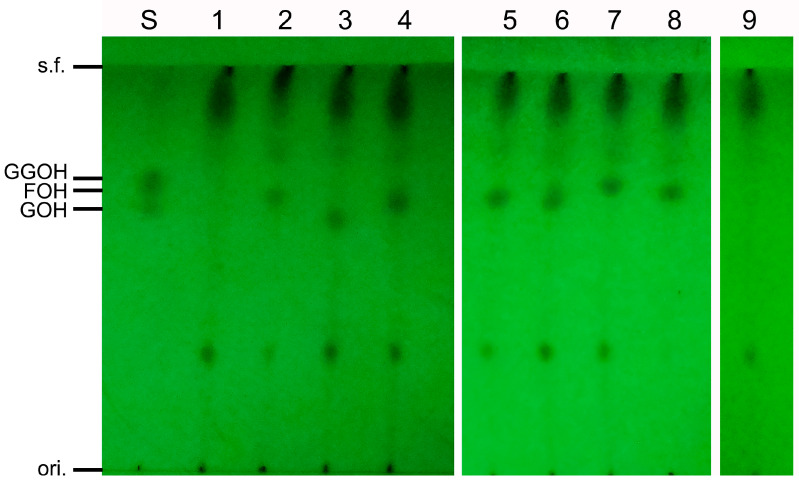
TLC analysis of in vitro enzyme activity reaction products of SpFPPS. (S: GOH, FOH and GGOH standard mix; 1, 3, 5 and 7: reaction system assay without SpFPPS protein using DMAPP, GPP, FPP and GGPP as substrates, respectively; 2, 4, 6 and 8: enzymatic reaction system containing SpFPPS protein with DMAPP, GPP, FPP and GGPP as substrates, respectively, 9: SpFPPS without substrates).

SpFPPS was incubated with IPP at apparent saturation and DMAPP or GPP as co-substrates to biocatalyze FPP; then, the products were hydrolyzed to the corresponding alcohols by incubating them overnight with an alkaline phosphatase [28], and no other chain length products were detected by TLC (Figure 4 Lane 2, 4). In addition, the product (FPP) visualized on TLC by iodine staining that was identified in the reaction between IPP and DMAPP was less intense compared to the stained products made in the reaction between IPP and GPP. The above results suggested that the *SpFPPS* gene encodes a functional farnesyl pyrophosphate synthase.

### 3.5. Design of Mutant SpFPPS

**Figure 5:**
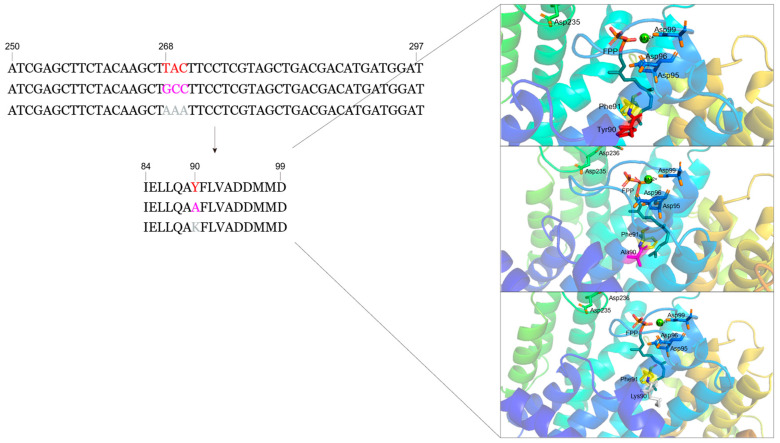
Visualization of FARM and the upstream fourth and fifth residues in the protein structure and sequences. Red represents SpFPPS, purple represents SpFPPS-Y90A, and gray represents SpFPPS-Y90K.

The tertiary structure of wt-SpFPPS and the predicted mutant proteins were visualized using Pymol, and the fourth and fifth amino acid residues upstream of FARM were labeled. It was observed that Y90 was located below the FPP chain, which was been identified in earlier works as hindering the extension of the FPP chain (Figure 5). In addition, Y is an aromatic amino acid with a large side-chain volume, and the 3D model presented for the mutated FPP may indicate that the replacement due to A and K renders the enzyme capable of synthesizing products longer than FPP (Figure 5). Therefore, we chose alanine (A) and lysine (K), which have small side-chain volumes, to replace Y and detected the effect of the replaced amino acids on the continued elongation of the FPP chain.

### 3.6. Construction, Expression and Purification of SpFPPS Mutants

Two mutant enzymes were constructed to determine whether the amino acid residue at the fifth position before FARM was associated with the length of the product chain. The mutant enzymes SpFPPS-Y90A and SpFPPS-Y90K were expressed in and purified from *E. coli* for in vitro assays (Figure S6A,B). In addition, the carbon chain length of the enzyme-catalyzed product varied with the reaction conditions in vitro. Therefore, the same enzyme assay conditions and methods used in the previous section were used for SpFPPS, SpFPPS-Y90A and SpFPPS-Y90K.

### 3.7. Mutant SpFPPS Enzymatic Activity Analysis

**Figure 6:**
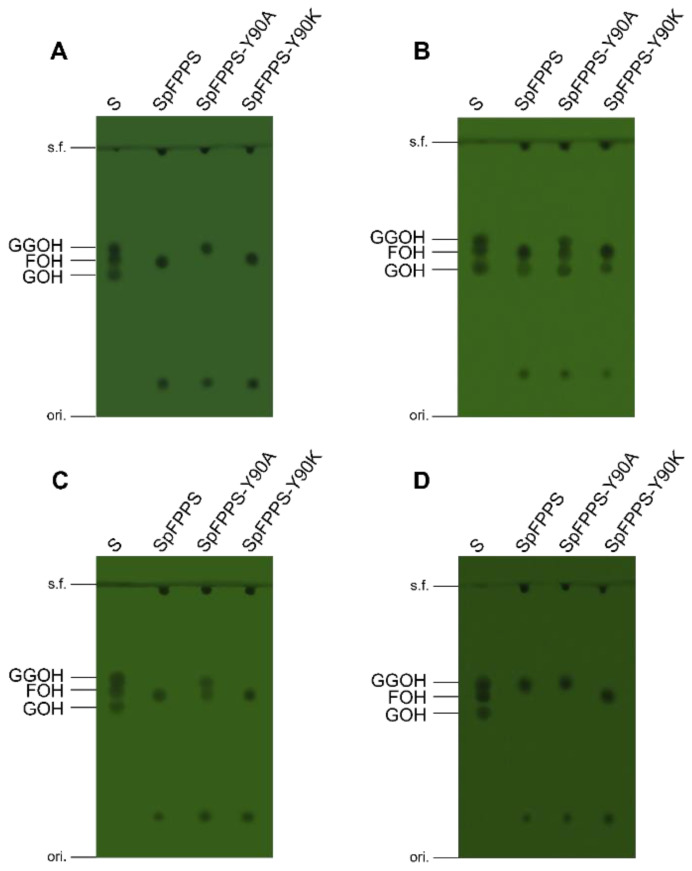
TLC analysis of in vitro enzyme activity reaction products of wild-type SpFPPS and mutant SpFPPSs. (**A**–**D**) Allyl diphosphate substrates were DMAPP, GPP, FPP and GGPP (S: GOH, FOH and GGOH standard mix).

The results for the in vitro SpFPPS and mutant protein enzyme activity are shown in Figure 6. SpFPPS, DMAPP, GPP and FPP were used separately as substrates with the IPP co-substrate. The product pool contained FPP, and GGPP was unaffected by SpFPPS, indicating that SpFPPS had the catalytic activity of FPP. The enzyme activity results for SpFPPS-Y90A showed that when DMAPP or GPP was used as a substrate, the products were FPP and GGPP, and that when FPP was used as a substrate, the product was GGPP. DMAPP, GPP, FPP and GGPP were converted by SpFPPS-Y90K extracts into a C15 allyl pyrophosphate that was detected as a C15 alcohol after dephosphorylation. This phenomenon indicated that the SpFPPS-Y90K protein hydrolyzes the substrate C20 GGPP to C15 FPP. Lao et al. identified an HpGGPP in *Haematococcus pluvialis*, which hydrolyzed C10 GPP or C15 FPP for C5 IPP to synthesize C20 GGPP, and this may be a mechanism for carotenoid accumulation for resistance to environmental stress [29].

The above results indicated that the substitution of the fifth amino acid upstream of FARM in SpFPPS changed the enzyme activity. According to the product distribution on TLC, SpFPPS-Y90A had FPPS/GGPPS bifunctional activity and synthesized C15 FPP and C20 GGPP simultaneously. SpFPPS-Y90K can hydrolyze GGPP or condense DMAPP or GPP with IPP to form C15 FPP.

## 4. Discussion

*S. pararoseus* is an unconventional fungus that produces a high yield of carotenoids, which are fat-soluble isoprenoids. Our previous study showed that it accumulates three carotenoids: β-carotene, torulene and torularhodin [16,20,30]. Humans cannot synthesize carotenoids by themselves, and the demand for natural anti-radiation and anti-aging products is increasing. Therefore, it is necessary to study the carotenoid synthesis pathway. The product FPP, catalyzed by FPPSs, is used as a constituent of primary metabolism compounds such as carotenoids and steroids [24]. Research on FPPSs has been pursued for many years, but the synthesis and mechanism of FPPS in *S. pararoseus* remain unclear.

We cloned a novel cDNA (*SpFPPS*) from *S. pararoseus*. TLC analysis showed that SpFPPS had activity in vitro. SpFPPS synthesized the sole product FPP regardless of the allylic substrate (DMAPP or GPP) that was utilized. The functions of FPP synthases had previously been used to study *Anthonomus grandis*, and the major product of AgFPPS was found to be FPP using DMAPP with IPP as substrates [31]. In fungus, FPP accounts for 75% of FPPS final products in *Saccharomyces cerevisiae* [32]. In *Xanthophyllomyces dendrorhous*, extracts of XdFPPS formed C10 and C15 allylic pyrophosphates [33]. Consistent with SpFPPS, they can use DMAPP or GPP as substrates, but in *Mycobacterium tuberculosis*, only geranyl diphosphate served as the MtFPPS acceptor [34]; the difference is that the enzyme activity product of SpFPPS is only FPP.

FPP provided a precursor for downstream synthesis of GGPP and other metabolic pathways, and most SCIPPSs catalyzed one specific product. However, some SCIPPSs have been reported to have two or even three enzyme activities (Table 1), and their products are also different. Perhaps the functional diversity of enzymes is to be attributed to their structural differences or is related to their long-term evolution. Furthermore, the reason why multifunctional enzymes prefer the above species to other organisms is still unclear and needs further exploration.

Comparing the CLD of SpFPPS with that of other species, it was shown that the structural characteristics of SpFPPS were consistent with the FPPSs of birds and humans (Figure 1A). According to the characteristics of CLD, SpFPPS was classified as a type I FPPS. The phylogenetic tree of SpFPPS with the FPPSs of other organisms obtained from the NCBI was constructed by the neighbor-joining method (Figure 1B). The tree had two branches, involving two independent non-homologous isoprenoid biosynthesis metabolic pathways: the mevalonate pathway (MVA) and the 1-deoxy-D-xylulose 5-phosphate/2-C-methyl-D-erythritol 4-phosphate pathway (DOXP/MEP) [43,44,45]. A branch of the phylogenetic tree was type I FPPSs, where the *Rhodotorula toruloides* FPPS was most similar to SpFPPS. SpFPPS had significant homology with *R. toruloides*, *Phaffia rhodozyma* and *Sanghuangporus baumii*, all of which belonged to the type I FPPSs. They had similar characteristics and mechanisms of chain length determination. Another branch contained type II and type III FPPSs. The FPPSs in *E. coli* and *Bacillus stearothermophilus* are type II FPPSs. Dhiman et al. identified a type III ω,E,E-farnesyl pyrophosphate synthase from *M. tuberculosis*, and they reported that the ω,E,E-farnesyl pyrophosphate synthase seems to be most similar to the type I GGPPSs from archaea, both containing the DDXXD motif [34]. The characteristic similarity between type I GGPPSs and type III FPPSs indicated that there is a probable evolutionary relationship between the two enzymes.

The length of the isoprenoid chain synthesized by SCIPPS is determined by the depth of the hydrophobic pocket inside the protein [46]. In NGR SpFPPS, an aromatic ring from Y forms the base of the pocket, which is located before FARM (Figure S4). Therefore, we hypothesized that the fifth amino acid residue upstream of FARM in SpFPPS was an aromatic amino acid (Y90), which is an important residue that blocks the extension of the FPP chain (Figure S4). In addition, the Y aromatic ring was replaced with a mutation of the smaller side chain to assess whether it would lead to the formation of longer chain products. Yan et al. [47] isolated and identified the GGPPS from NGR, which was classified as a type III GGPPS according to its CLD characteristics; the fifth amino acid upstream of FARM was A. Previous studies reported that the FPPS in *Toxoplasma gondii* has FPPS/GGPPS bifunctional activity. Li et al. [48] mutated the upstream fifth cysteine (C) to Y, which converted the enzyme into absolute FPPS to generate only FPP; they determined that the C at the fourth position is vital for TgFPPS bifunctionality, and the C corresponded to Y in SpFPPS. Therefore, we replaced the 90th Y with A and K, and the mutant recombinant proteins SpFPPS-Y90A and SpFPPS-Y90K were obtained.

The in vitro enzyme activity results showed that IPP reacted with the substrate DMAPP or GPP, that the enzyme activity product was FPP and that no other chain length product was produced in the enzyme activity system containing SpFPPS. The mutants showed different properties. SpFPPS-Y90A produced FPP and GGPP with DMAPP or GPP as the substrate and had FPP/GGPPS bifunctional enzyme activity. Interestingly, the only product of SpFPPS-Y90K was FPP. However, when C20 GGPP was used as the substrate, the product of SpFPPS-Y90K was C15 FPP. Therefore, we considered that the mutation changed the structure of the protein so that it had the function of hydrolyzing the substrate. The reaction mechanism needs further exploration. As the biosynthetic pathway of *S. pararoseus* isoprenoids is elucidated more fully, it will be possible to further explore the detailed mechanism of FPPSs and to construct engineering strains.

## 5. Conclusions

In this study, we cloned the *SpFPPS* of *S. pararoseus*, identified the function of its gene expression products, explored the relationship between the SpFPPS protein structure and its evolution, and tracked the enzymatic activity mechanism of the SpFPPS protein deeply. An obtained mutant bifunctional FPPS/GGPPS showed the biochemical properties of individual FPPSs and GGPPSs from other species. However, other organisms typically contain characteristic FPPSs and GGPPSs, which provide a reference for the next step of gene modification to regulate enzyme activity and for further exploration of the carotenoid synthesis pathway in *S. pararoseus*, providing a molecular basis for further study of the regulation of the pigment synthesis yield and the pigment synthesis pathway mechanism in *S. pararoseus*. Further studies are needed to define and elucidate SpFPPS and its encoded enzyme features precisely.

## Figures and Tables

**Table 1:** Functional diversity of isoprenyl diphosphate synthases.

Enzymes	Organism	Products	Reference
FPPS	*Zea mays* L.	FPP, GGPP	[35]
FPPS	*Myzus persicae*	GPP, FPP	[36]
GPPS	*Phalaenopsis bellina*	GPP, FPP	[37]
GPPS	*Picea abies* (Norway spruce)	GPP, FPP, GGPP	[38]
GPPS	*Picea abies*	GPP, GGPP	[39]
GPPS	*Lithospermum erythrorhizon*	GPP, FPP	[40]
FPPS	*Plasmodium falciparum*	FPP, GGPP	[41]
FPPS	*Toxoplasma gondii*	FPP, GGPP	[42]
GGPPS	*Haematococcus pluvialis*	GPP, FPP, GGPP	[29]
SpFPPS-Y90A	*S. pararoseus*	FPP, GGPP	This study

## Data Availability

All relevant data can be found within the article and the Supplementary Materials and are available from the corresponding author upon request.

## Supplementary Materials

The following supporting information can be downloaded at: https://www.mdpi.com/article/10.3390/cimb46040195/s1, Figure S1: Electrophoresis results of *SpFPPS* gene in *S. pararoseus* NGR. (M: marker; 1: genomic DNA; 2: cDNA); Figure S2: Sequence comparison of cDNA and genomic DNA of *S. pararoseus* NGR *SpFPPS*; Figure S3: Bioinformatic analysis of the *S. pararoseus* NGR SpFPPS protein; Figure S4: Model of the three-dimensional structure of the *S. pararoseus* NGR SpFPPS; Figure S5: SDS-PAGE analysis of recombinant protein; Figure S6: SDS-PAGE analysis of protein; Table S1: Primers used in this study; Table S2: Plasmids used in this study and their main characteristics.
