# Supplementary material for: The Property of a Key Amino Acid Determines the Function of Farnesyl Pyrophosphate Synthase in Sporobolomyces pararoseus NGR
Source: Curr Issues Mol Biol. 2024 Apr 3;46(4):3108–21. doi: 10.3390/cimb46040195 (PMC11048977; doi:10.3390/cimb46040195)
Supplement: Supplementary file 1 [file cimb-46-00195-s001.zip › Supplyment information.pdf]

Supplyment information

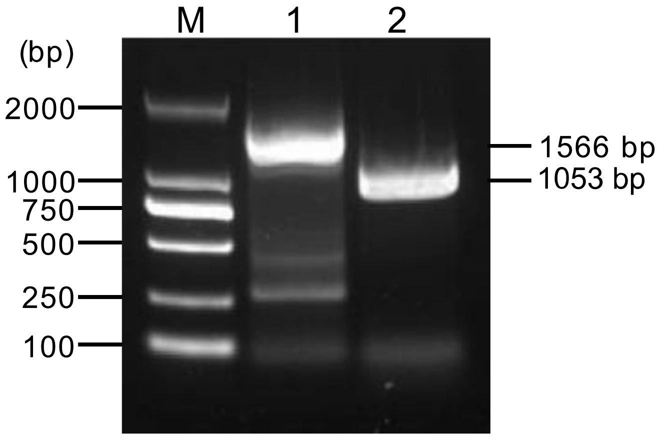

Figure. S1 Electrophoresis results of *SpFPPS* gene in *S. pararoseus* NGR. (M: Marker; 1: genomic DNA; 2: cDNA)

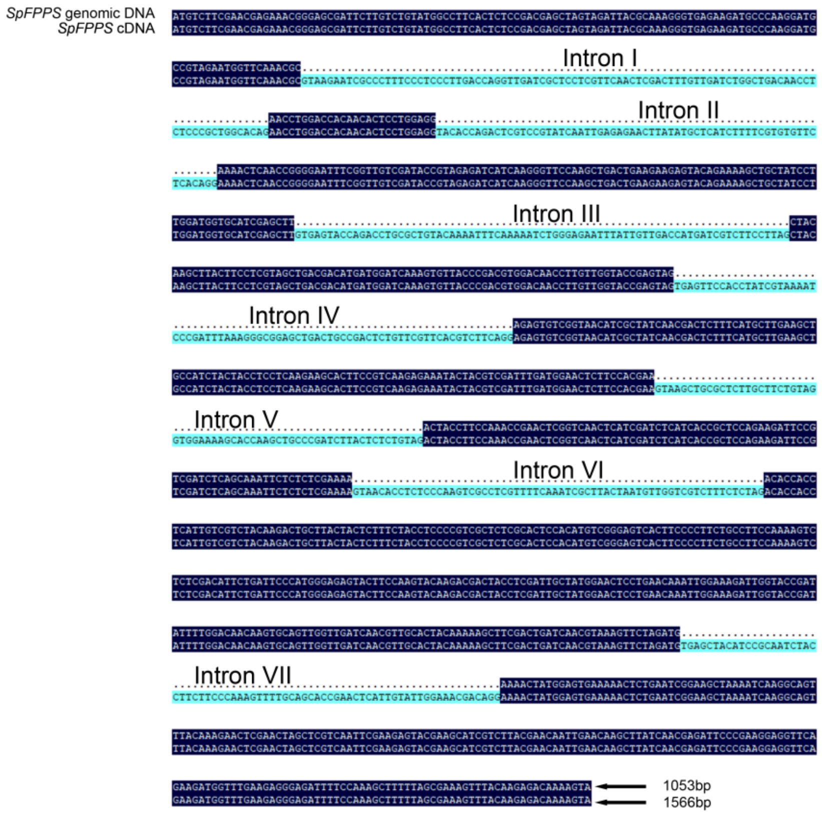

Figure S2 Sequence comparison of cDNA and genomic DNA of *S. pararoseus* NGR *SpFPPS*.

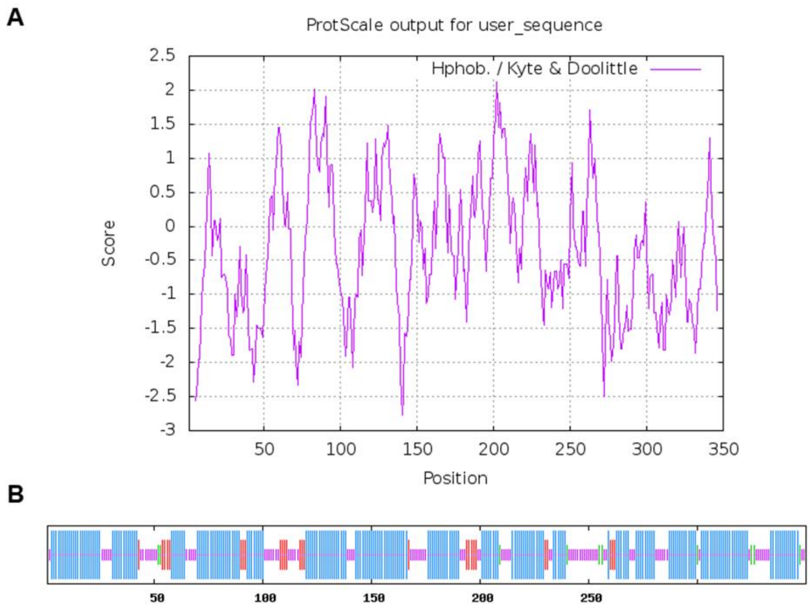

Figure. S3 Bioinformatics analysis of the *S. pararoseus* NGR SpFPPS protein.

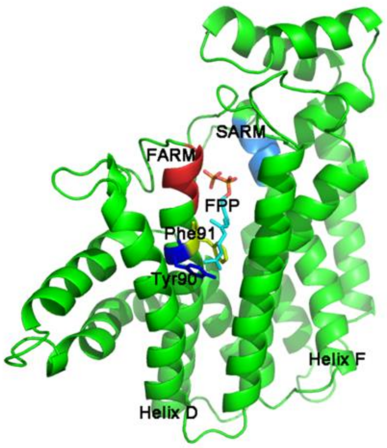

Figure S4. Model of the three-dimensional structure of the *S. pararoseus* NGR SpFPPS.

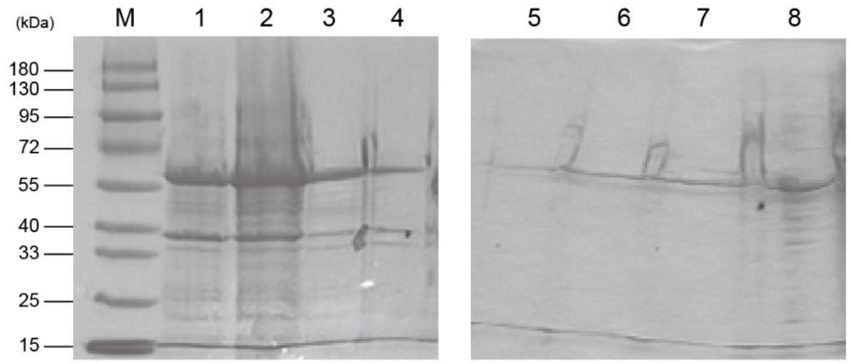

Figure. S5 SDS-PAGE analysis of recombinant protein.

SDS-PAGE analysis of pET32a-SpFPPS induced to be expressed at different temperatures. (M: Marker; 1-4: Supernatants of BL21(DE3) strains carrying plasmid pET32a-SpFPPS after fragmentation induced at 28, 26, 23 and 20°C; 5-8: precipitate after fragmentation of BL21(DE3) strain carrying plasmid pET32a-*SpFPPS*.

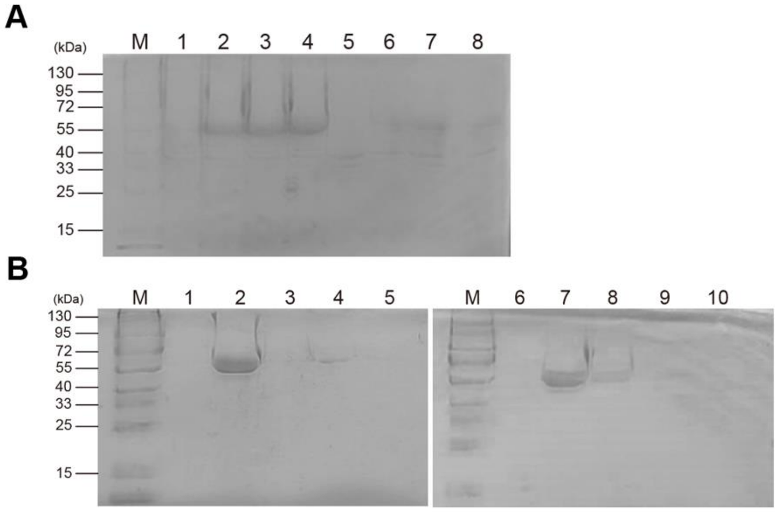

Figure. S6 SDS-PAGE analysis of protein.

A. SDS-PAGE analysis of protein pET32a, pET32a-SpFPPS, SpFPPS-Y90A and SpFPPS-Y90K (M: Marker; 1-4: supernatants of BL21(DE3) strains carrying plasmid pET32a, pET32a-*SpFPPS*, *SpFPPS-Y90A* and *SpFPPS-Y90K* after fragmentation; 5-8: precipitation of BL21(DE3) strain carrying plasmid pET32a、pET32a-*SpFPPS*, *SpFPPS-Y90A* and *SpFPPS-Y90K* after fragmentation)

B. Purification of protein SpFPPS-Y90A and SpFPPS-Y90K

(M: Marker; 1-5: SpFPPS-Y90A was sequentially eluted using Elution Buffer; 6-10: SpFPPS-Y90K was sequentially eluted using Elution Buffer)

Table S1. Primers used in this study.

Primers	Sequences (5′-3′)	Restriction Enzymes
FPPS-F	CGGGATCCATGTCTTCGAACGAGAAACGG	*Bam*HI
FPPS-R	CGAGCTCCTACTTTTGTCTCTTGTAAACTTTCG	*Sac*I
FPPS-Y90A-Fm	ACAAGCTGCCTTCCTCGTAG	-
FPPS- Y90A-Rm	CTACGAGGAAGGCAGCTTGTA	-
FPPS- Y90K-Fm	ACAAGCTAAATTCCTCGTAGCTG	-
FPPS- Y90K-Rm	CTACGAGGAATTTAGCTTGTAGAAGC	-

Note: The restriction sites were underlined, the shadow mark was replaced base. All primers were synthesized by Shanghai Sangon Inc.

Table S2. Plasmid used in this study and their main characteristics.

Plasmids	Relevant Characteristics	Source or Referance
pMD18-T vector	Amp^r	Takara, Beijing, China
pET-32a (+) vector	Amp^r	This study
pMD18-T-*SpFPPS*	Amp^r	This study
pET32a-*SpFPPS*	Amp^r	This study
pMD18-T-*SpFPPS-Y90A*	Amp^r	This study
pET32a-*SpFPPS-Y90A*	Amp^r	This study
pMD18-T-*SpFPPS-Y90K*	Amp^r	This study
pET32a-*SpFPPS-Y90K*	Amp^r	This study

Note: Amp^r: ampicillin resistant.
