# Supplementary figures and images for: The Property of a Key Amino Acid Determines the Function of Farnesyl Pyrophosphate Synthase in Sporobolomyces pararoseus NGR
Source: Curr Issues Mol Biol. 2024 Apr 3;46(4):3108–21. doi: 10.3390/cimb46040195 (PMC11048977; doi:10.3390/cimb46040195)

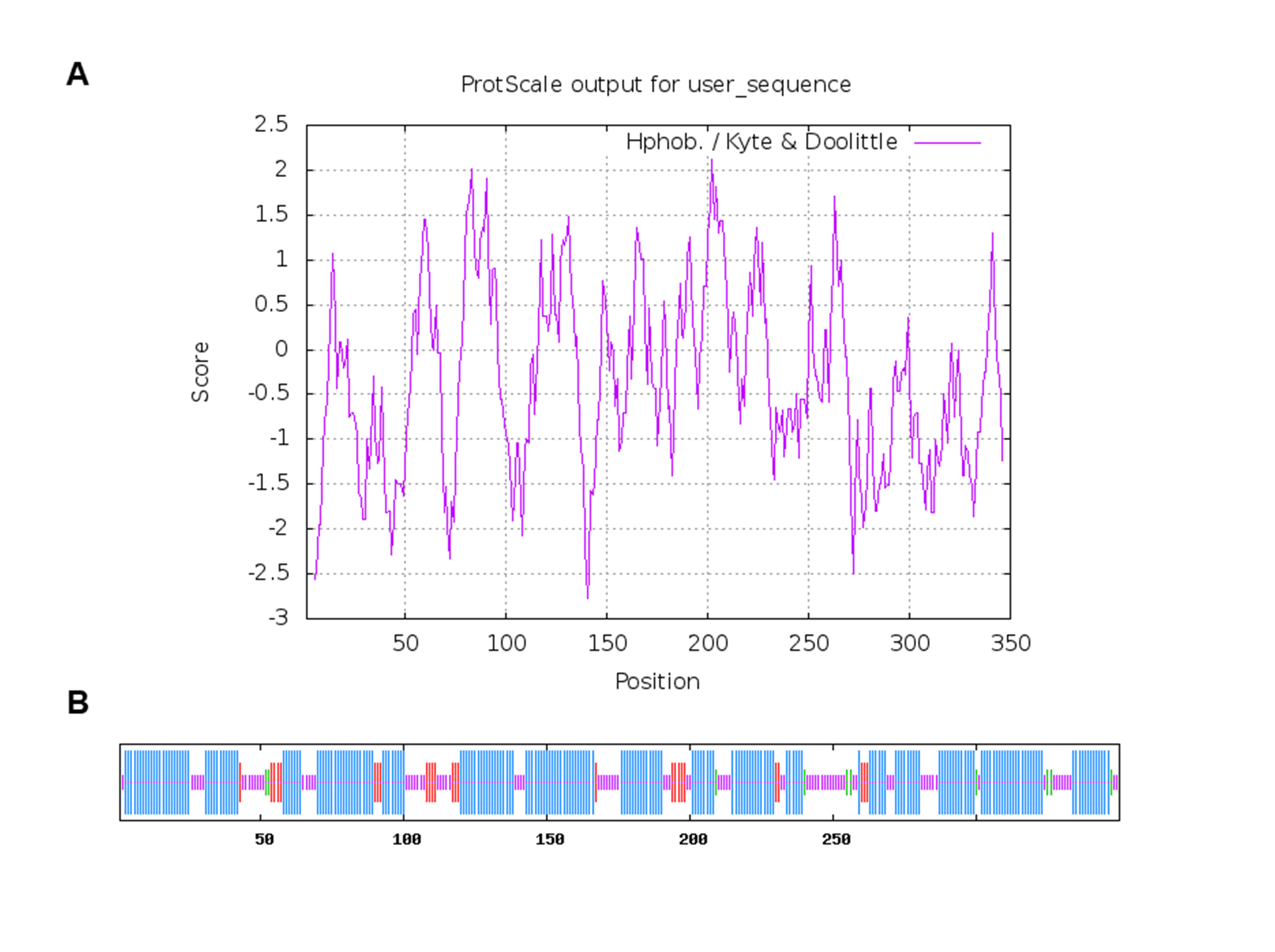

Supplement: Supplementary file 1 [file cimb-46-00195-s001.zip › Figure S3.tif]

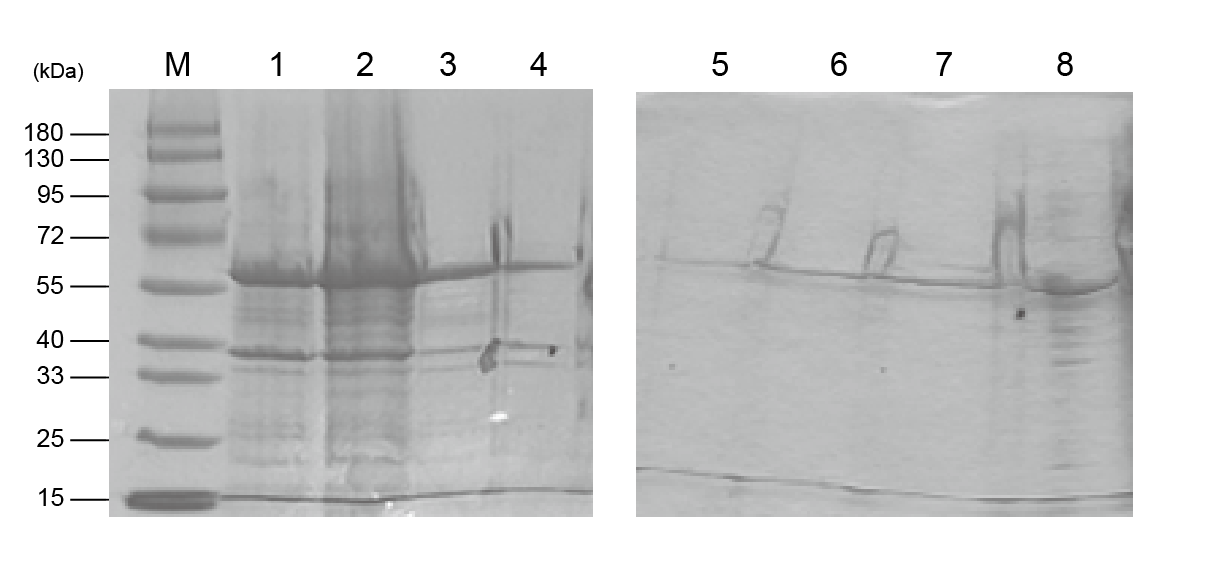

Supplement: Supplementary file 1 [file cimb-46-00195-s001.zip › Figure S5.tif]

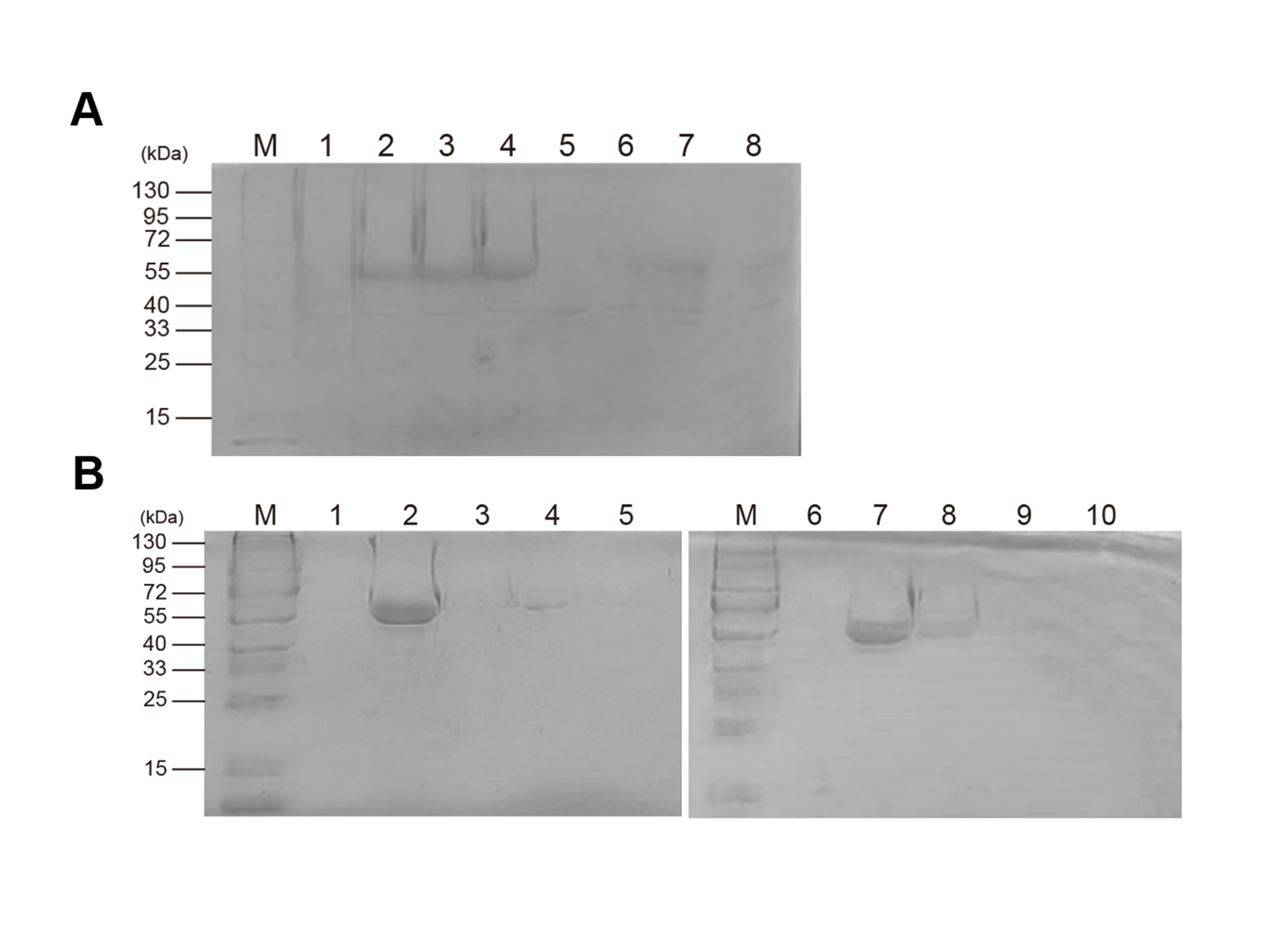

Supplement: Supplementary file 1 [file cimb-46-00195-s001.zip › FigureS6.tif]
